# Organ preservation using a photosynthetic solution

**DOI:** 10.1186/2047-1440-1-2

**Published:** 2012-04-24

**Authors:** Ippei Yamaoka, Takeshi Kikuchi, Tomohiro Arata, Eiji Kobayashi

**Affiliations:** 1 Otsuka Pharmaceutical Factory, Inc, 115 Kuguhara, Tateiwa, Muya-cho, Naruto, Tokushima, 772-8601, Japan; 2 Center for Development of Advanced Medical Technology, Jichi Medical University, 3311-1 Yakushiji, Shimotsuke, Tochigi, 329-0498, Japan

**Keywords:** Photosynthesis, Donation after cardiac death, Pancreas, Transplantation, Respiratory failure, Warm ischemia, Rat

## Abstract

**Background:**

Organs harvested from a body lapsing into circulatory deficit are exposed to low O_2_/high CO_2_, and reach a critical point where original functionality after transplantation is unlikely. The present study evaluates the effect of respiratory assistance using *Chlorella* photosynthesis on preservation of the rat pancreas from the viewpoint of donation after cardiac death (DCD).

**Methods:**

Gas was exchanged through the peritoneum of rats under controlled ventilation with or without *Chlorella* photosynthetic respiratory assistance. A gas permeable pouch containing *Chlorella* in solution was placed in the peritoneum and then the space between the pouch and the peritoneum was filled with an emulsified perfluorocarbon gas carrier. Rat DCD pancreases procured 3 h after cardiac arrest were preserved for 30 min in a cold or mildly hypothermic environment or in a mildly hypothermic environment with photosynthetic respiratory support. The pancreases were then heterotopically transplanted into rats with STZ-induced diabetes.

**Results:**

Levels of blood oxygen (PaO_2_) and carbon dioxide (PaCO_2_) increased and significantly decreased, respectively, in rats with mechanically reduced ventilation and rats given intraperitoneal photosynthetic respiratory support when compared with those without such support. Transplantation with DCD pancreases that had been stored under photosynthetic respiratory support resulted in the survival of all rats, which is impossible to achieve using pancreases that have been maintained statically in cold storage.

**Conclusion:**

Respiratory assistance using photosynthesis helps to improve not only blood gas status in the event of respiratory insufficiency, but also graft recovery after pancreas transplantation with a DCD pancreas that has been damaged by prolonged warm ischemia.

## Background

Clinical outcomes have considerably improved for patients after organ transplantation, which has become a standard procedure in most developed countries. However, improved clinical outcomes have led to a global shortage of organs, which has encouraged unethical organ trafficking [1]. The value of organs harvested from donation after cardiac death (DCD) donors has been reassessed and applied in the clinical setting [2]. However, oxygen depletion results in the accumulation of acidic products that causes irreversible damage to harvested DCD organs [3]. Static cold storage is a traditional method of organ preservation that arrests cellular metabolism and prevents ATP depletion. However, this does not avoid reperfusion injury to the organ induced by the elevated temperature after transplantation.

Plants and eukaryotic microalgae have permanent intracellular photosynthetic organelles (chloroplasts) for autotrophic growth. On the other hand, various metazoans such as colonial ascidians, molluscs, sponges and cnidarians form an intra- and intercellular algal symbiotic relationship with intracorporeally integrated photosynthetic prokaryotes from the environment or an inherited product [4,5]. Incorporated chloroplasts maintain photosynthetic ability and the photosynthetic products are utilized for saccharide synthesis or the anabolism of nitrogen by host organisms [6].

We initially attempted to create a symbiotic relationship between rats and a photosynthetic microalga, and removed CO_2_ from and supplied O_2_ to the bodies of rats with respiratory insufficiency. We then developed complementary gas exchange between animal respiration and plant photosynthesis to support the respiration of DCD organs and consequently transplanted DCD pancreases obtained at 3 h after cardiac arrest into rats. This remains impossible after traditional static cold preservation. The present findings should produce a paradigm shift in the concept of traditional preservation and remarkably extend the likelihood of transplantation with organs from DCD donors.

## Materials and methods

### *Chlorella* photosynthesis

Oxygen produced by *Chlorella* photosynthesis was initially examined under various conditions. Chlorella suspensions (*Chlorella vulgaris*; 1 × 10^10^ cells/mL; Chlorella Industry, Fukuoka, Japan) diluted 0-, 6.25-, 12.5-, 25-, 50- and 100-fold were tested under 0, 0.2, 0.6 and 1.8 g/dL of NaHCO_3_. Photosynthetic abilities were also compared under lighting supplied by halogen lamps and by a custom-designed light-emitting diode (LED). We then determined that the optimal conditions for the following experiments from the perspectives of O_2_ production and CO_2_ elimination were *Chlorella vulgaris*, 2.2 × 10^8^ cells/mL in 0.6 g/dL NaHCO_3_. Seven LED lights with a luminous flux of 70,000 lux stabilized the temperature of the photosynthetic solution.

### Measurements of O_2_ bubbles produced by *Chlorella* photosynthesis

The sizes of O_2_ bubbles produced *in vitro* were characterized using scattered laser diffraction that provides the distribution of particle size (Spraytech, Malvern, UK). We then estimated gas exchange capacity between the photosynthetic solution and a carrier emulsion containing 30% perfluorocarbon (PFC) via an O_2_-permeable porous membrane (OTP-35H, provided by Otsuka Techno Corporation, Tokushima, Japan). The oxygen concentrations in the PFC carrier were also compared between photosynthesis and oxygen bubbling.

### Rat model of respiratory failure

The committee for animal experiments at the Research and Development Department of Otsuka Pharmaceutical Factory Inc. approved the following experimental procedures. Adult male Sprague-Dawley rats (Charles River Japan, Yokohama, Japan) weighing 490–600 g were housed under constant humidity and temperature (22 ± 2°C) under a 12:12-h light-dark cycle. An incision of about 5 cm was cut in the rat abdomen under intravenous (i.v.) anesthesia with sodium pentobarbital (initially 50 mg/kg, supplemented with 25 mg/kg/h) and then the abdominal wall was opened to expose the incision to LED illumination. A vinyl tube (inner diameter, 0.5 mm) was inserted into the right carotid artery for blood sampling, and then a 14-gauge plastic tube was placed into the trachea to control ventilation. Heparin (100 IU/kg) was injected i.v. followed by a muscle relaxant (Mioblock; 2 mg/kg i.v. initially and supplemented with 1 mg/kg at 75 min after induction). The rats were immediately connected to a respirator delivering ventilation with 10 mL/kg of room air. A temperature controller (NS-TC, Neuroscience, Inc., Tokyo, Japan) maintained the rectal temperature at 37°C throughout the experiment.

The ventilation rate was maintained at 70 strokes/min for the first 15 min and a 10 × 10 cm porous pouch containing 100 mL/kg of photosynthetic *Chlorella vulgaris* 2.2 × 10^8^ cells/mL in 0.6 g/dL NaHCO_3_ was placed in the rat peritoneal cavity. The rats were then assigned to the following groups (n = 6 per group): immediate illumination via LED to start photosynthesis (light, L) or not (Dark, D; Controls) in the presence or absence of PFC emulsion [L-PFC (+) or L-PFC (−) and D-PFC (+) and D-PFC (−), respectively]. At 45 min after implantation with a pouch containing *Chlorella*, the 30% PFC-emulsion diluted with normal saline was channelled between the abdominal wall and the O_2_-permeable membrane at a dose of 50 mL/kg in the L-PFC (+) and D-PFC (+), but not in the L-PFC (−) and D-PFC (−) groups. Oxygen and carbon dioxide levels in the heparinized blood in these rats were monitored using Model 348 pH/blood gas analyzer (Chiron Diagnostics, Halstead, UK) at 15-min intervals.

### Pancreatic transplantation 3 h after cardiac arrest

Donor male LEW rats (Charles River Laboratories Japan Inc., Yokohama, Japan) weighing 200–300 g were fasted from the day before transplantation. Recipient rats were intravenously administered with 50 mg/kg of streptozotocin (Sigma-Aldrich Japan, Tokyo, Japan) in citric acid buffer 3 days before transplantation. All rats had glycemic levels of ≥300 mg/dL.

Cardiac arrest was induced in donor rats by the inhalation of excess diethyl ether and then the rats remained at room temperature for 3 h. The pancreas was removed via laparotomy along with the spleen and the common bile duct was ligated. The sheath of an 18 G Surflow cannula (Terumo, Tokyo, Japan) was inserted into the abdominal aorta of the pancreas, which was then irrigated with 200 μL of 10% heparin in normal saline at room temperature. The DCD rat pancreases were then preserved in 1.5 g/dL of hemoglobin derived from bovine blood (Sigma, Tokyo, Japan) in an organ preservation solution (ETK, Otsuka Pharmaceutical Factory Inc., Tokushima, Japan) for 30 min at 4°C (cold), 22°C (mildly hypothermic) or at 22°C with photosynthetic respiratory support. Photosynthesis proceeded in the *Chlorella* solution (50 mL) inside the gas-permeable pouch that was immersed in photosynthetic preservation solution at PaO_2_ >300 mmHg.

Recipient rats were anesthetized by the inhalation of diethyl ether and the right side of the neck was incised. The right jugular vein and carotid artery were separated, and then cuffs (Terumo, Tokyo, Japan) comprising the sheaths of the 20 G and 16 G Surflow catheters were attached to each, respectively. The abdominal aorta was anastomosed to the carotid artery, the portal vein was anastomosed to the jugular vein and the pancreas graft was reperfused. Thereafter, the spleen was resected from the pancreas graft, and the neck skin was closed with a continuous suture. Normal saline (5 mL) was intradermally administered immediately after surgery, blood was collected 4 h later and 2 mL of 25% glucose was intraperitoneally administered thereafter.

### Tissue O_2_ measurements

Tissue oxygen (PaO_2_) levels were determined using an O_2_ electrode system (Oxy Lab; Oxford Optronix Ltd., Oxford, UK). Organs isolated from rats 3 h after cardiac arrest were immersed in organ preservation solution (ETK, Otsuka Pharmaceutical Factory Inc., Tokushima, Japan) containing 1.5 g/dL hemoglobin (Hb-ETK) from bovine blood (Sigma, Tokyo, Japan) at 22°C under photosynthesis or O_2_ bubbling or at 4°C without either treatment. The *Chlorella* was substituted with saline and O_2_ inspired inside the bag was bubbled.

### Statistical analysis

Values for each group are presented as means ± SD. Differences among the groups were analyzed by two-way repeated measures and one-way ANOVA or *χ*^2^ test, and Tukey’s post hoc tests if appropriate. Statistical significance was set at *p* < 0.05.

## Results and discussion

### Complementary gas exchanges between rats and *Chlorella*

**Figure 1:**
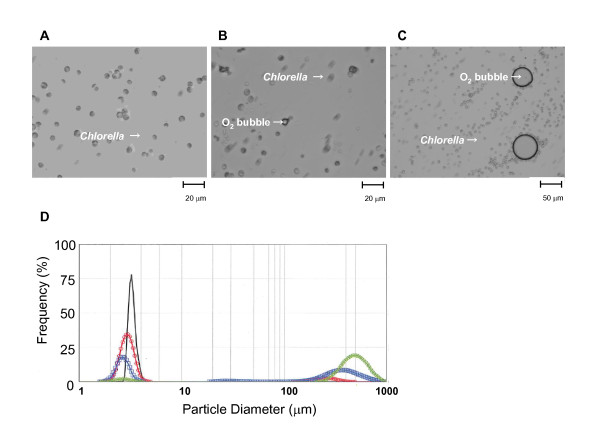
**Light microscope images of oxygen (O**_**2**_**) bubbles generated by photosynthesis in*Chlorella*(*A-C*). D:** Distribution of frequencies of volume occupied by each particle in photosynthetic solution before (black line) and at 5 (red), 10 (blue), and 20 (green) min after light-emitting diode illumination. The peak before illumination indicates *Chlorella* particles (black line).

We first measured the O_2_ bubbles produced during *Chlorella* photosynthesis. Air bubbles of various sizes (2–1000 μm) were found in the photosynthetic solution (Figure 1). Particles (2–4 μm) of O_2_ bubbles were detected on the surface of *Chlorella* 5 min after starting photosynthesis (Figure 1D). The gas gradually increased and O_2_ bubbles of about 700 μm in diameter dominated the particles that appeared in the photosynthetic solution 20 min after LED illumination. The photosynthetic reaction precludes the need for specialized foaming equipment to generate oxygenated micro-bubbles or nano-bubbles [7]. We compared the flow of gases generated by *Chlorella* photosynthesis and by O_2_-bubbling through the gas-permeable membrane. Oxygen partial pressure (PaO_2_) gradually increased to 300–600 mmHg within 30 min of either solution being placed on the other side of the oxygen production system, indicating that O_2_ can penetrate the membrane (data not shown).

**Figure 2:**
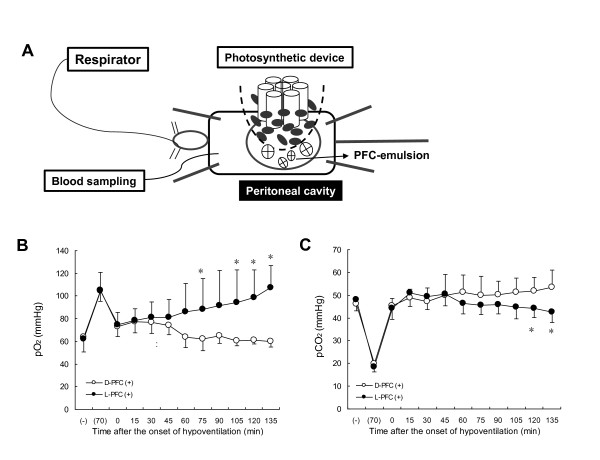
**Schema of photosynthetic respiratory support through the peritoneum (A).** Arterial blood gas measurements (PO_2_, **B**; PCO_2_, **C**) in anesthetized rats under hypoventilation with () or without photosynthesis (○); (−) and (70), periods before and 15 min after 70 strokes/min of ventilation, respectively. Space between rat abdomen and gas-permeable pouch filled with 30% perfluorocarbon emulsion 45 min after starting hypoventilation (0 min). Values are means ± S.D (n = 6). * Significant difference (*P* < 0.05).

Some silicone oils and fluorocarbon solutions equilibrated with O_2_ gas support mammalian respiration through the lungs [8]. A gas-permeable pouch containing a photosynthetic solution of *Chlorella* implanted into the peritoneum of anesthetized rats with a mechanically reduced respiratory rate was illuminated using an LED light to start photosynthesis. Figure 2A summarizes our *in vivo* model of respiratory failure with a low frequency of respiration. The PaO_2_ decreased in the blood and PaCO_2_ increased after reducing the ventilation rate. However, the PaO_2_ of the rats under photosynthetic respiratory assistance gradually increased, whereas the PaCO_2_ decreased after filling the space between the abdominal wall and the gas-permeable membrane with the perfluorocarbon (PFC) gas-carrier (Figure 2B and C). Photosynthesis did not affect PaO_2_ and PaCO_2_ unless PFC was injected into the space (data not shown). These results show that adding an aqueous gas carrier to fill the space between a mammalian abdomen and *Chlorella* within a gas-permeable environment results in complementary gas exchange between different species. Studies of artificial lungs have examined sites other than lungs, particularly direct injection of O_2_ gas to the peritoneum [9] or the peritoneal perfusion of liquids containing O_2_[10]. We believe that we have pioneered the use of a photosynthetic microalga to remove of CO_2_ from, and supply O_2_ to the whole body of rats with respiratory insufficiency.

### Respiratory assistance to DCD pancreases using *Chlorella* photosynthesis

**Figure 3:**
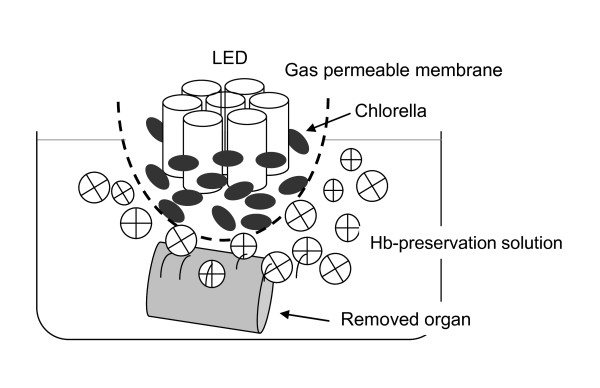
**System for photosynthetic respiratory assistance of harvested organs.** Pancreases isolated from donor rats at 3 h after cardiac arrest are placed in preservation solution containing 1.5 g/dL bovine hemoglobin. An oxygen permeable pouch containing *Chlorella* in 50 mL of solution (0.6 g/dL NaHCO_3_) is immersed in 50 mL of preservation solution illuminated with an immersible custom-designed device comprising seven LED lights (70,000 lux) for 30 min. Oxygen generated from *Chlorella* photosynthesis is transferred to preservation solution via permeable film.

**Figure 4:**
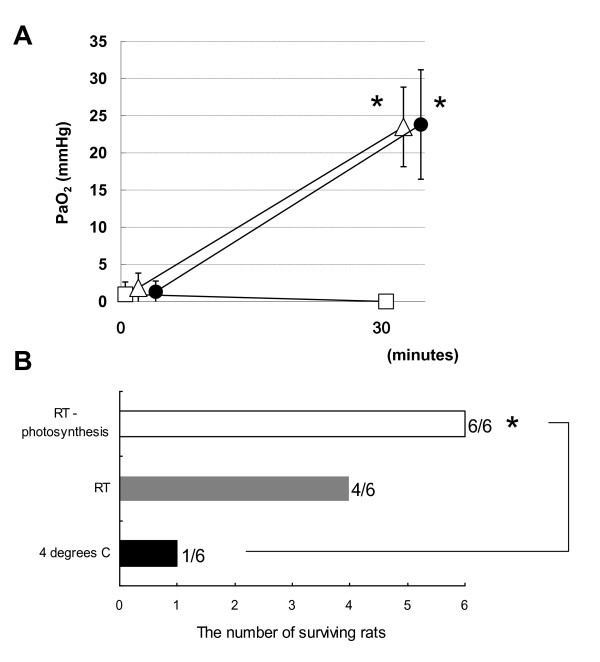
**Changes in partial O**_**2**_**(mmHg) of rat pancreas isolated 3 h after cardiac arrest and immersed in preservation solution containing bovine hemoglobin (A).** Pancreas immersed in preservation solution containing bovine hemoglobin without further manipulation at 4°C, □; respiratory assistance through photosynthesis at 22°C, △; oxygen bubbling at 22°C, . Both respiratory assistance systems were placed on other side of the pancreas via a gas permeable membrane. Values are means ± SD; n = 3. * Significant difference from storage without further manipulation (*p* < 0.05). **(B).** Effect of photosynthetic respiratory assistance on survival rates of rats ectopically transplanted with pancreases obtained from rats at 3 h after cardiac arrest. Survival rates of recipient rats for up to 7 days after transplantation were compared after 30 min of immersion in preservation solution at 4°C or at room temperature (RT; 22°C) with or without photosynthesis before transplantation. * Significant difference from storage at 4°C (*p* < 0.05).

Respiratory assistance using photosynthesis might also be applicable to the preservation or revival of O_2_- depleted and CO_2_- enriched organs with circulatory failure harvested from DCD donors. We established a respiratory support system to reverse this process in organs (Figure 3). The PaO_2_ of the rat pancreas retrieved 3 h after cardiac arrest was essentially 0 mmHg after cold static preservation (Figure 4A). The PaO_2_ increased in the DCD pancreas assisted by respiration using photosynthesis for 30 min at 22°C. The PaO_2_ also increased over a period of 30 min via direct bubbling from an oxygen cylinder in the positive control organ for oxygenation. These results indicated that preservation under photosynthetic respiratory assistance causes oxygenation in organs even after 3 h of cardiac arrest in a fashion similar to that of general oxygenation via direct O_2_ infusion. The mechanisms underlying the penetration of organs with O_2_ remains to be determined, but 15% oxygenation of the total volume of the pancreas achieved using the two layer method (TLM) is sufficient to maintain ATP levels in islet cells damaged by warm ischemic injury [11]. Therefore, oxygenation by photosynthesis would improve the function of organs exposed to warm ischemia and recover grafts exposed to prolonged warm ischemia.

We then evaluated the outcomes of providing respiratory assistance to DCD organs after transplantation. Pancreases removed from rats 3 h after cardiac arrest were preserved under either traditional static cold storage (4°C) or under mildly hypothermic (22°C) conditions with or without respiratory assistance, and then transplanted into rats with STZ-induced diabetes mellitus. We previously evaluated the ATP contents of pancreases obtained from Luc Tg rats [12] at 1 or 5 h after cardiac arrest. Since the luciferase gene with the Rosa 26 promoter is chromosomally integrated and expressed in all organs of this rat strain, changes in ATP concentrations in harvested organs can be determined from chips of sliced tissues obtained from these rats. The ATP contents of the pancreas substantially decreased at 1 h after cardiac arrest but decreased to minimal levels at 5 h thereafter. We also clarified that rats transplanted with a pancreas at 3 h after cardiac arrest immediately die, whereas those transplanted within 1 or 2 h thereafter do not. These data imply that reducing pancreatic ATP levels determines the feasibility of transplantation [13].

Five of the six rats that received organs that were traditionally preserved in a cold environment for 30 min after isolation died at 3 to 5 h after transplantation, whereas all rats that received organs preserved under photosynthetic respiratory assistance survived for over 1 week (Figure 4B). One rat each that was transplanted with an organ preserved at 22°C without photosynthetic support died within 5 and 7.5 h and the survival rate did not significantly differ from that of animals transplanted with organs that had been traditionally preserved at 4°C.

Hyperglycemia and hypoglycemia (glucose > 600 and < 20 mg/dL, respectively) were identified in two of three animals at 4 h after transplantation with traditionally preserved organs. In contrast, none of the rats transplanted with organs preserved under photosynthesis developed hyperglycemia ≥180 mg/dL. However, three of six rats transplanted with organs preserved at 22°C without photosynthesis became hyperglycemic (glucose concentration ≥ 180 mg/dL). Plasma insulin levels were not examined in the present study, but early hyperinsulinism caused by irreversible ischemia/reperfusion injury of grafts indicates poor outcomes such as death attributable to hypoglycemia [14,15]. We postulated that the cause of the death after transplantation is attributable to ischemic damage that causes early high insulin release from a non-functioning pancreas and subsequent hypoglycemia.

Static cold storage is currently the most popular form of preservation in routine clinical practice. Although simple and effective, whether this method can prevent the deterioration of organ quality is questionable when increasing numbers of organs are becoming harvested from older, more marginal donors, including DCD donors [16]. In fact, even a short period of cold exposure (0.5 h) significantly deteriorates the favourable impact of mildly hypothermic immersion. In another set of experiments, we constructed a system in which photosynthesis proceeded in a preservation solution maintained at 4°C, and then the pancreas was transplanted into diabetic rats (n = 6). Three rats died but the one survivor had aberrant glucose control (glucose > 600 mg/dL). These results imply that preservation using respiratory assistance requires an optimal temperature. The viability of organ donors from harvest to transplantation is crucial for maintaining optimal graft function and impacts survival rates [17]. These results indicate that the outcomes of pancreatic transplantation would improve if oxygen generated by photosynthesis were provided to tissues with an increased oxygen demand caused by preservation at room temperature.

Preservation using photosynthesis might have several limitations when applied to the clinical setting or to larger animals. The question would arise as to whether this technique could provide adequate oxygenation and deeper O_2_ penetration into solid and/or large organs as in TLM, in which some discrepancies in its benefits are apparent between small and large animal experimental models [18,19]. Furthermore, even in small animal models, oxygen delivery is inadequate for organs with dense and robust capsules such as the liver and kidneys [20]. Therefore, combination with other methods such as automated perfusion with immersion might improve the oxygenation of solid and larger organs [21], although this would be rather complex and difficult for routine application.

Some populations of eukaryotes can form symbiotic connections [6,22]. Artificial manipulation has never enabled a living entity or its zooblast to coexist with photosynthetic microorganisms or its organelles. Furthermore, concomitant gas-exchange between plants and animals has not, to the best of our knowledge, been reported. Therefore, we discovered that complementary respiration through photosynthesis can be achieved by artificial symbiosis between a microalga and a mammal or its organs.

## Competing interests

E. K. is a special advisor to Otsuka Pharmaceutical Factory Inc. The other authors declare no competing financial interests.

## Authors’ contributions

IY designed the study, performed the study *in vivo* and *in vitro*, collected data, performed statistical analysis, interpreted the findings and drafted the manuscript. TK performed the study *in vivo* and collected data. TA performed the study *in vitro* and collected data. EK designed and coordinated the study and helped to draft the manuscript and interpret data. All authors read and approved the final manuscript.
